# A peer support dietary change intervention for encouraging adoption and maintenance of the Mediterranean diet in a non-Mediterranean population (TEAM-MED): lessons learned and suggested improvements

**DOI:** 10.1017/jns.2023.2

**Published:** 2023-01-30

**Authors:** Katherine M. Appleton, Claire T. McEvoy, Christina Lloydwin, Sarah Moore, Patricia Salamanca-Gonzalez, Margaret E. Cupples, Steven Hunter, Frank Kee, David R. McCance, Ian S. Young, Michelle C. McKinley, Jayne V. Woodside

**Affiliations:** 1 Department of Psychology, Faculty of Science and Technology, Bournemouth University, Fern Barrow, Bournemouth BH12 5BB, UK; 2 Centre for Public Health, Queen's University Belfast, Grosvenor Road, Belfast BT12 6BJ, UK; 3 Functional Nutrition, Oxidation and Cardiovascular Disease Group (NFOC-SALUT), Facultat de Medicina i Ciències de La Salut, Universitat Rovira i Virgili, Sant Llorenç, 21, 43201 Reus, Spain; 4 Regional Centre for Endocrinology and Diabetes, Belfast Health and Social Care Trust, Grosvenor Road, Belfast BT12 6BA, UK

**Keywords:** Dietary change, Mediterranean diet, Mixed methods, Peer support, Process evaluation

## Abstract

Peer support interventions for dietary change may offer cost-effective alternatives to interventions led by health professionals. This process evaluation of a trial to encourage the adoption and maintenance of a Mediterranean diet in a Northern European population at high CVD risk (TEAM-MED) aimed to investigate the feasibility of implementing a group-based peer support intervention for dietary change, positive elements of the intervention and aspects that could be improved. Data on training and support for the peer supporters; intervention fidelity and acceptability; acceptability of data collection processes for the trial and reasons for withdrawal from the trial were considered. Data were collected from observations, questionnaires and interviews, with both peer supporters and trial participants. Peer supporters were recruited and trained to result in successful implementation of the intervention; all intended sessions were run, with the majority of elements included. Peer supporters were complimentary of the training, and positive comments from participants centred around the peer supporters, the intervention materials and the supportive nature of the group sessions. Attendance at the group sessions, however, waned over the intervention, with suggested effects on intervention engagement, enthusiasm and group cohesion. Reduced attendance was reportedly a result of meeting (in)frequency and organisational concerns, but increased social activities and group-based activities may also increase engagement, group cohesion and attendance. The peer support intervention was successfully implemented and tested, but improvements can be suggested and may enhance the successful nature of these types of interventions. Some consideration of personal preferences may also improve outcomes.

## Introduction

The Mediterranean diet (MD) has been widely identified as a healthy dietary pattern contributing to a favourable health status^(1–4)^. Many reviews of observational studies now confirm a role for MD in reducing cardiovascular disease (CVD) risk, outcomes and mortality^(1–8)^, trials demonstrate causal associations^(1–3,5,6,9,10)^, and, while the most consistent and robust evidence for health benefits have been observed in relation to CVD^(1–4)^, benefits have also been found for all-cause mortality^(1,6,8)^, obesity^(1–3)^, metabolic syndrome^(1,2,11)^, type 2 diabetes^(1–3)^, some cancers^(2,4,12,13)^, and have been suggested for other health outcomes^(1–4)^.

Representing the dietary pattern traditionally consumed among populations bordering the Mediterranean Sea^(4,6)^, the MD is characterised by a high consumption of minimally processed seasonal, fresh and locally grown fruits, vegetables, legumes, beans, nuts and seeds, a moderate consumption of dairy products, eggs, fish, seafood and poultry, and a low consumption of red meat, processed meat and ultra-processed foods. It is also characterised by the use of extra virgin olive oil as the main source of fat^(2,4,6)^. Individual components of the MD, such as extra virgin olive oil and nuts, have well-documented health benefits^(2,4,7,14,15)^, but in recent years, particular attention has been paid to the overall food combination or dietary pattern; an approach that reflects the consumption of foods as opposed to nutrients, that captures the synergistic effects of individual nutrients and foods, and may allow increased detection of effects from constituents that may otherwise be small^(4,5,16,17)^.

In clinical trials, successful dietary change towards MD consumption has been achieved, predominantly with resource-intensive interventions, offered by health professionals and usually in parallel with food provision^(1–3,5,6,9,10)^. However, the transferability and acceptance of MD to populations outside of the Mediterranean region may remain a challenge^(2,6,7,11,18,19)^. Several studies point to numerous barriers to adopting and adhering to the MD in these populations^(20–27)^. Important barriers, as reported in Australian^(26)^, UK^(20,22,23,25)^ and US^(21)^ populations, are knowledge of the required foods^(20–23,26)^; access to and affordability of these foods^(22,23,25,26)^; the acceptability of these foods and the acceptability of a diet with only minimal consumption of certain other foods, such as red meat^(20–22,25,26)^; the time and skills required for food shopping, preparation and organisation^(21–23,25,26)^; and motivation to maintain the diet within a physical, social or cultural environment that may not provide good support^(20,22,23,25,26)^. In the Northern Irish population specifically, we^(24)^ and others^(27)^ found similar concerns based on the acceptability, including the healthiness, of certain foods, the cost and availability of relevant foods, the knowledge, time or cooking skills that may be required, the suitability of the MD^(24)^ or a MD-style intervention^(27)^ for the Northern Irish climate, culture and dietary traditions and the challenge of changing established eating patterns^(24,27)^. In non-Mediterranean populations, olive oil and legume consumption is often low, and meat consumption is often higher than recommended for the MD^(18,19,28)^.

The resource-intensive nature of some interventions may also be prohibitive in some situations. One alternative to an intervention led by a dietitian or other health professional involves the use of peer support. Peer support is defined as ‘the provision of emotional, appraisal, and informational assistance by a created social network member who possesses experiential knowledge of a specific behaviour or stressor and similar characteristics as the target population, to address a health-related issue of a potentially or actually stressed focal person’^(29, p. 329)^. Peer support can be provided on an individual- or group-basis, directly from peer supporters or more remotely, e.g. via the telephone^(30)^, and interventions using peer support have resulted in improved health behaviours^(31–33)^. An absence of benefit and challenges in implementing this type of intervention, however, are also reported^(29,30,34–36)^.

We recently developed a group-based peer support MD intervention for use in a Northern Irish population^(24,37,38)^, and investigated its effects in a pilot trial^(39,40)^. The intervention was designed based on theory, qualitative work and in conjunction with the target population, to address population-specific barriers. Full details are given elsewhere^(24,37,38)^. Briefly, the intervention consisted of eleven group sessions, delivered by two trained peer supporters, over a 12-month period. Peer supporters were recruited and trained in advance of intervention implementation, using a bespoke training programme. Groups involved up to ten participants who met in a convenient location within their community. Each group session lasted up to 2 h and included a brief (10–15 min) MD and behavioural education component, designed to provide a focus for group discussion. The group topics included: ‘health benefits of a MD’, ‘changing fat intake’, ‘eating more wholegrain’ and ‘eating a seasonal MD’. Written educational materials were developed specifically for the intervention, to include an MD information booklet (explaining MD, the health benefits and general tips for following MD), suggested meal plans, seasonal recipe books and shopping lists, and a personal planner to facilitate dietary goal-setting and self-monitoring. Personal body weight and blood pressure measurements were available in each session, with feedback offered by the peer supporter, and practical food demonstrations (via food tasting) were included in four sessions. Participants were also encouraged to maintain contact with other group members and peer supporters between sessions to promote support and group cohesion.

The pilot trial compared the effectiveness of the peer support intervention with two other MD interventions for impacts on MD score and various health risk markers. The Trial to Encourage Adoption and Maintenance of a MEditerranean Diet (TEAM-MED) was a 12-month pilot parallel group randomised controlled trial implemented in Northern Ireland, aiming to evaluate the feasibility of a community-based peer support intervention, compared with a dietician-led intervention and a minimal support intervention (which served as a control group), in a non-Mediterranean population at high risk of CVD^(40)^. The study protocol and detailed methodology are published elsewhere^(39,40)^. Briefly, participants were recruited and included in the study if they were aged 40 years or older, had low adherence to MD (≤3 points on a locally adapted 14-item MD score (MDS)), a BMI > 27 and < 45 kg/m^2^ and a combination of risk factors, that, according to the Joint British Societies CVD risk prediction tables, placed them at an estimated multifactorial risk of CVD ≥ 20% in 10 years^(41)^. Participants were randomised to one of three intervention arms: peer support intervention; dietitian-led intervention and a minimal support intervention; at a ratio of 1:1:1. The three interventions varied in the intensity and nature of support provided to encourage the adoption and maintenance of dietary behaviours consistent with a MD. Dietary behaviours included: increased consumption of wholegrains, fruit, vegetables, fish (especially oily fish), legumes, unprocessed nuts, olive oil and/or rapeseed oil and olive oil-based spreads; reduced consumption of red and processed meat; and moderate alcohol consumption (if already consumed). Rapeseed (canola) oil was allowed alongside olive oil, given the similar fatty acid composition, reduced cost and it is locally produced in Northern Ireland. The peer support intervention was intended to provide group-based support from trained lay peers. The dietitian-led intervention was designed to provide support from a health professional using a combination of individual- and group-based sessions to mimic the resource-intensive interventions that have previously shown success elsewhere^(9)^, and included limited MD food provision. The minimal support intervention was intended to provide only minimal support and included provision only of the written educational materials. All study participants received the information booklet and practical information for following a MD (meal plans, recipe books and shopping lists). The peer support group additionally received a personal planner.

As part of the pilot trial, we sought to investigate the feasibility of running the intervention, to better understand how the intervention worked in practice and how it could be improved. This process evaluation was pre-specified in advance of study conduct^(39)^. Pre-specified aims were to test the validity of the theoretical model underpinning the peer support intervention; evaluate the training and support provided to peer supporters to deliver the intervention; determine fidelity of implementation and acceptability of the intervention; assess outcome data collection processes within the pilot trial, including those to explore mediators and moderators of MD adherence and cost-effectiveness; and explore reasons for withdrawal for study participants and peer supporters recruited to deliver the peer support intervention^(39)^. The present paper aimed to evaluate the peer support intervention, from the perspectives of those delivering and receiving it. This includes consideration of the peer supporter training and support; intervention fidelity and acceptability; acceptability of the data collection processes for the trial; and any reasons for withdrawal. Data relevant to theoretical processes, such as those exploring mediators and moderators to MD adherence, will be reviewed elsewhere. The study was conducted according to the guidelines laid down in the Declaration of Helsinki and all procedures involving human participants were approved by the Office for Research Ethics Committees Northern Ireland (HSC RECA; ref 13/NI/0152). Written informed consent was obtained from all participants.

## Methods

Peer supporter training and support were assessed through questionnaires and interviews with peer supporters. Intervention fidelity was assessed through observation. Intervention acceptability and the acceptability of data collection processes were assessed through interviews with study participants, and a final evaluation questionnaire. Reasons for withdrawal, where applicable, were assessed by interview.

### Peer supporter questionnaires

#### Perceptions of the peer supporter training

A 16-item feedback questionnaire designed for the study was administered at the end of the training. All aspects of the training were rated on a five-point scale from ‘strongly disagree’ to ‘strongly agree’, and subsequently scored from 0 to 4 respectively, where higher scores denote more positive perceptions.

#### Impacts of the training on knowledge, skills and confidence to deliver the TEAM-MED intervention

A 5-item feedback questionnaire was designed for the study and administered at the start and end of the 2-d training for peer supporters. This questionnaire asked peer supporters to rate their knowledge (3 items), confidence (1 item) and skills (1 item) to deliver the intervention as ‘poor’, ‘fair’, ‘good’ or ‘excellent’. Responses were subsequently scored from 0 to 3, respectively, where higher scores denote higher impacts.

### Peer supporter interviews

Peer supporters were also contacted after the end of the study to request their participation in a semi-structured interview, aiming to explore their experiences in the study. Topic guides were created and tested with two researchers prior to use. A sample topic guide is given in the Supplementary Material.

### Observations

Intervention fidelity was assessed using observations by the study researchers of the intervention as provided. Aspects of the intervention under scrutiny were: time between participant screening and intervention start; number of intervention sessions run; number of participants attending each intervention session, and two intervention sessions (at Month 6/7 and Month 12) were observed in detail using direct in-person observation. Checklists were used to structure these direct observations, allowing study researchers to report: the inclusion (or not) of twenty-three different elements of the intervention that were expected in each session; participation by trial participants (or not) in eleven different elements of the intervention that were provided in each session; an assessment of group cohesion on a sliding scale from 0 to 10 and any additional notes. A copy of the checklist is given in the Supplementary Material.

### Study participant interviews

Participants were contacted after the end of the study to request their participation in a semi-structured interview about their experience in the TEAM-MED study. Topic guides were created, as above, tested with two researchers and finalised for use with participants.

### Study participant questionnaires

#### Final evaluation

An 8-item feedback questionnaire was also administered at the end of the trial to all participants following the peer support intervention. Various aspects of the trial and intervention were rated on a five-point scale from ‘strongly disagree’ to ‘strongly agree’. Responses were subsequently scored from 0 to 4, respectively, where higher scores denote more positive perceptions.

### Data analysis

Questionnaire data were analysed using IBM SPSS Statistics 28 to produce descriptive and summary information. Observations are reported as recorded in real-time. Interviews were recorded and transcribed verbatim. Nvivo 10 was used to conduct a Framework analysis of the data; an analysis chosen to facilitate a deductive analysis approach given that the process evaluation research questions were pre-determined. The process of analysing the transcripts involved: familiarisation with transcripts; developing and applying an analytical framework; charting the data into the framework and interpretation^(42)^. Qualitative quotes are provided throughout the results section. Additional quotes are provided in the Supplementary Material.

## Results

### Peer supporter training and support

Thirteen peer supporters were recruited from the general public and from local community networks, volunteer websites and health centres. Peer supporters were required to have an MDS score > 3 and/or a CVD risk < 20% with the intention that they had already made changes to their lifestyle or reduced their CVD risk, and were to be a lay participant or community health worker or volunteer. No peer supporter could have had a clinical CVD event or other health condition, and had to be committed and motivated to complete the training and deliver the intervention. Early work with our target population^(37)^ identified that a peer supporter would ideally ‘have successfully made changes towards a MD, have expert dietary knowledge’, such that ‘group members would feel the peer supporter is like them and wanted to make similar dietary changes to them’^(p. 8)^,  … and ‘should be empathetic, encouraging and have a good sense of humour, plus they should have personal experience of eating a MD, good knowledge of health and budgeting, and strong communication and listening skills’^(37, p. 8)^. Our criteria for recruiting peer supporters were intended to ensure that peer supporters were similar to the ideal identified in the earlier work. Being a similar gender, age or from the same location were not considered to be important^(37)^, but our recruitment strategies also ensured a sufficient level of background literacy and dietary knowledge. Potential peer supporters attended an interview conducted by two members of the research team. This process was used to determine their capacity and commitment to undergo the required training, their attitudes towards dietary change and their compassion and understanding of the difficulties that people with high CVD risk may encounter when striving to achieve dietary change. Previous work with groups along with appropriate inter-personal, communication and group facilitation skills were also considered desirable for the peer supporter role.

Peer supporters were trained over two full days. The training programme and accompanying manual was designed and produced by four researchers from the study team (CME, SEM, MMK, JVW), to include details of the peer supporter role and responsibilities; education about MD, common barriers to following an MD and to dietary change, behaviour change techniques and some underlying theory on dietary change; and skills related to group facilitation, social support and taking physical measurements. Training was delivered by two researchers (CME, SEM) with the aid of a dietitian qualified in dietary behaviour change and group facilitation skills.

All peer supporters attended the training. The training was considered by the peer supporters to be relevant, comprehensive and easy to understand (mean (sd) score = 3⋅8 (0⋅5) out of 4), with a good mix of learning activities and breaks (mean score = 3⋅8 (0⋅4)), was supported by a useful, clear and well-organised manual (mean score = 0⋅9 (0⋅4)) and was provided by facilitators who were thought to be knowledgeable, well prepared and responsive (mean score = 4⋅0 (0⋅1)). Training also resulted in changes in knowledge, confidence and skills to deliver the intervention (see Table 1).

**Table 1.** Mean scores (0–3) in knowledge, confidence and skills before and after training in the peer supporters (*N* = 13)

	Before training	After training
Knowledge of MD	1⋅2	2⋅6
Knowledge of TEAM-MED intervention	0⋅7	2⋅5
Knowledge of TEAM-MED intervention resources	0⋅5	2⋅5
Confidence to deliver intervention	1⋅0	2⋅2
Skills to deliver intervention	1⋅0	2⋅3

Support for the peer supporters was also given following the training. Each peer supporter was provided with a manual containing the information from the training, and the resources that would be needed for running the intervention. Additionally, peer supporters could contact the research team at any stage, and members of the research team were in regular contact with the peer supporters to discuss and evaluate intervention sessions.

Seven peer supporters provided qualitative comments at the end of the trial. High questionnaire scores for the training were backed up by positive comments:

*It was good. The resources that we had were very good, the folder and the props that we had were very good, and the training went through all of that*. PS3, Group 2

*It was very a good mixture of theory based with the resources, the paper resources that you had to use with each of the groups. It was all very clearly laid out and there was a good combination of the theory, actually doing the workshops you'd be getting the group to do and then feeding back what you thought of the workshops and the processes. It was very interactive sort of learning through doing and learning with your other peer supporters and getting to know them as well.* PS1, Group 4

Some suggestions for additional support were also given, e.g. for additional materials or greater depth for some of the topics covered. There was also a delay between training and intervention provision, and two peer supporters suggested that the delay between training and intervention start may have reduced their enthusiasm for the intervention and resulted in the need to revise before taking on the peer supporter role:

*I suppose sometimes maybe it was just some of the topics maybe were a bit light in detail and you found you had to do a bit more research yourself and reading beforehand just to make sure that … just go and Google wholegrain or whatever, just to get a bit more information, a bit of depth behind, because, at the end of the day, you have to spend two hours with the group talking about it and you maybe felt that you needed a wee bit more information in the pack or on each topic, it would have been helpful.* PS1, Group 4

*Once I did start I had to really reread everything, which wasn't a bad thing. But I think I would have liked maybe a quicker start from the initial training.* PS2, Group 2

Refresher training for peer supporters to reduce the impact of this delay did reduce concerns, but due to prior commitments, only four of the peer supporters could attend; all others receiving a summary email.

Positive comments from peer supporters were also given on implementing the intervention. Peer supporters discussed enjoying their role, enjoying seeing group members make changes towards MD and learning about the MD themselves:

*I suppose that's what motivates me a lot, to try and help more people be healthier, live healthier lifestyles. So yeah, it was very positive. We met some very interesting people, and I suppose I learnt quite a bit about Mediterranean eating and Mediterranean lifestyle and tried to incorporate a bit of it.* PS1, Group 4

*I suppose seeing them get to know each other as a group and to see how their individual journeys, how they kind of progressed and the changes that they were able to implement. So that was really interesting. And just to see, over the course of the year, how their enthusiasm didn't really wane at all, like they stayed really, really, sort of really keen and really focused, which, it was really nice to see that.* PS2, Group 1

Additional mutual support may also have been beneficial:

*It would have been nice to have a bit of support maybe from another mentor, especially, funny enough, the last session, which is the one I would have thought would be the easiest, but that was the one that you had to recap everything and because I had forgotten my folder, I forgot the questions [laughing] But it would have been nice to have a little bit more support on that.* PS3, Group 3

One peer supporter withdrew from the role part-way through the intervention. This withdrawal was a result of changes in personal circumstances and commitments, but further details could not be gained.

### Intervention fidelity

Twenty-seven (36 %) participants were randomised to the peer support intervention as part of the pilot study, although only twenty-six participants attended the baseline session. Peer support groups were created as participants were screened and entered the study, to result in the set-up of four peer support groups. Groups ranged in size to include from 4 to 10 participants, and were held in community venues, in and around Belfast, Northern Ireland. Two peer supporters were allocated to run all sessions for each group as a pair. Peer supporters were selected for each group based on location, and with an aim to create pairs of peer supporters with both strong dietary knowledge and strong group facilitation skills.

Key participant characteristics for each of the four peer support groups are given in Table 2. These varied across the groups to some degree. Notably, the number of people in each group varied from 4 to 10, and Groups 2 and 3 were predominantly composed of females, while Groups 1 and 4 were predominantly composed of males. Fewer participants in Group 3 did most of the cooking in their household. Mean MD score at screening was also higher in Groups 1 and 2.

**Table 2.** Descriptive characteristics of participants in the individual peer support groups

	PSG 1 (*n* = 6)	PSG 2 (*n* = 4)	PSG 3 (*n* = 6)	PSG 4 (*n* = 10)
Sex				
Female *n* (%)	2 (33⋅3)	3 (75⋅0)	4 (66⋅7)	2 (20⋅0)
Male *n* (%)	4 (66⋅7)	1 (25⋅0)	2 (33⋅3)	7 (70⋅0)
Mean (sd) age at baseline (year)	59⋅2 (4⋅1)	52⋅0 (2⋅9)	58⋅5 (6⋅4)	55⋅1 (7⋅4)
Mean (sd) full-time education at baseline (year)	12⋅3 (1⋅4)	14⋅3 (2⋅1)	14⋅0 (3⋅0)	13⋅4 (3⋅1)
Relationship status, *n* (%):				
Married/Co-habiting	4 (66⋅7)	2 (50⋅0)	5 (83⋅3)	4 (40⋅0)
Single/Widowed/Separated/Divorced	2 (33⋅3)	2 (50⋅0)	1 (16⋅7)	4 (40⋅0)
Most cooking in household, *n* (%):				
Participant	4 (66⋅7)	4 (100⋅0)	2 (33⋅3)	4 (40⋅0)
Spouse/partner/child	0 (0⋅0)	0 (0⋅0)	2 (33⋅3)	2 (20⋅0)
Shared	2 (33⋅3)	0 (0⋅0)	2 (33⋅3)	2 (20⋅0)
Mean (sd) BMI at screening (kg/m^2^)	37⋅5 (5⋅7)	35⋅2 (1⋅6)	35⋅0 (5⋅4)	35⋅0 (4⋅6)
Mean (sd) Med Diet score at screening	2⋅3 (0⋅8)	2⋅5 (0⋅6)	1⋅8 (1⋅3)	1⋅6 (1⋅0)

#### Time between participant screening and intervention start

The intervention began following recruitment of a number of participants from the same geographical location and agreement on a meeting venue and time. These logistical considerations resulted in a delay in beginning the intervention for some participants, and this differed between groups, such that for Group 1, the mean (sd) delay was 85 (47) days; for Group 2, this was 117 (40) days; for Group 3, this was 133 (110) days and for Group 4, the mean (sd) delay was 109 (93) days.

#### Number of intervention sessions run

All intervention sessions were delivered, resulting in the conduct of eleven sessions per peer support group. All sessions for Groups 1, 2 and 4 were run with two peer supporters. For Group 3, one peer supporter experienced personal difficulties during the study, resulting in some absence and eventual replacement with an alternative peer supporter for the group.

#### Number of participants attending each intervention

**Fig. 1.**
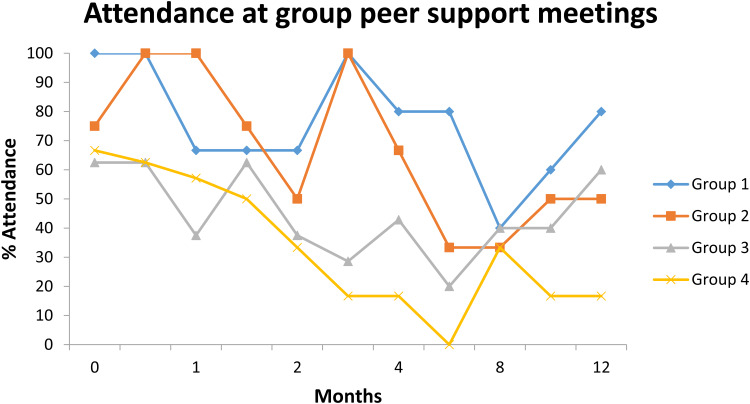
Attendance at each of the peer support group meetings over the intervention.

Attendance at the peer support meetings was not 100%, even at the start, and dropped over the 12 months of the intervention (see Fig. 1).

#### Detailed observation of two intervention sessions

Elements of intervention provision under the control of the peer supporters were marked as present/absent, out of a possible 23, in two sessions, one at Month 6/7, one at Month 12. Participation by attendees in elements of the intervention was also assessed with a score out of 11, and group cohesion was given a score out of 10. Ratings are given in Table 3.

**Table 3.** Number of intervention elements present out of 23, participation by attendees out of 11, and group cohesion score out of 10, for each group, at month 6/7 and month 12

Month	Assessment of the Session	PSG 1	PSG 2	PSG 3	PSG 4
6/7	Intervention elements (out of 23)	20	20	13	21
	Attendee participation (out of 11)	4	7	7	3
	Group cohesion (out of 10)	8	8	7	7⋅5
12	Intervention elements (out of 23)	22	17	15	14
	Attendee participation (out of 11)	8	4	5	3
	Group cohesion (out of 10)	8	4	5⋅5	2

Observations of these sessions demonstrated both positive and negative aspects, as given in the researcher's notes taken at the time:

Month 6/7:

- PSG 1: Peer supporters contributed equally to the session; were calm, open, approachable, supportive, positive and used humour; discussed solutions, stayed on topic. Could be more enthusiastic.
- PSG 2: Peer supporters did not really work together – one was positive, part of the group, gave advice from her viewpoint, the other differentiated herself from the group, was more of a teacher figure, less focused. Both were very encouraging. Some recapping from previous meetings, some recipe sharing by participants, lots of laughter.
- PSG 3: Only one peer supporter in attendance. She was relaxed, friendly, one of the group, but she did not have a structured approach to the meeting, could have been more organised and perhaps more encouraging. Peer supporter did not seem to have contacted participants to remind them about the session, some cynicism.
- PSG 4: Peer supporters were open, co-operative, welcoming, came with posters of session dates and their phone numbers, brought seasonal vegetables, olive oil, nuts and seeds, looked over the autumn recipe book, discussed bringing foods for next session, lots of recipe sharing, but did not discuss goals.

Month 12:

- PSG 1: Peer supporters worked well together, both very encouraging, positive, very open about problems, provided examples from personal lives, challenged participants to try a recipe between sessions.
- PSG 2: One peer supporter seemed much more supportive than the other, both seemed discouraged by lack of attendance, both sympathised more than encouraged. One peer supporter was accusative about failures on holiday.
- PSG 3: Only one peer supporter in attendance, meeting not very structured, generally positive attitude. No measurements offered, slightly stressful start, due to bad traffic and other activities in the building. One participant brought in a recipe.
- PSG 4: Peer supporters seemed to have lost enthusiasm, reported themselves finding it difficult to eat MD. There was a focus on difficulties.

### Intervention acceptability

#### Intervention sessions

Comments on the intervention sessions were positive. Participants reported benefits from the intervention sessions, and enjoyed taking part:

*I was more than happy with the sessions, they were interesting, sometimes challenging.*

*I've really enjoyed being part of the study. It has been life changing for me; it definitely has. The way in which my lifestyle is very different and the way that I eat and even the way that I think about food now.* Ppt 008, Group 3

*I thought it was great, it was really, really helpful. I'm just sorry that they started to spread them out so far apart. I enjoyed going to the classes and we all got on very well and exchanged recipes and tips and things.* Ppt 026, Group 2

Participants were generally positive particularly about the group-based nature of the sessions. Support and encouragement were gained from the peer support group in the form of sharing knowledge, such as where to buy key MD foods at low cost; sharing ideas, such as how to include MD foods that they found less palatable into their diets, sharing recipes and actual cooked dishes and increasing motivation, e.g. by discussing future goals and sharing successes:

*It was good just to learn about the difference between wholegrain and all those sort of things, because sometimes you hear all this talk and you feel a bit silly because you don't understand. It wasn't like that; any time that you were in the group you didn't feel as if you were asking a silly question.* Ppt 012, Group 1

*If you said “oh I've tried that,” or especially if somebody had achieved a goal of, like I say, when he had said he had walked, and we were all “oh that's fantastic!” and “imagine doing that!” Or equally too, if somebody had tried something for the first time, even if they didn't like it, we'd say “well at least you tried it.” So it would have been very encouraging.* Ppt 008, Group 3

Suggestions to improve the supportive nature of the group were also provided, both by participants and peer supporters to include more personal, social and interactive activities, and more communication between group meetings:

*a Facebook page or something like that that's relatively quick and easy to set up, that again your sharing tips, hints, successes, “did you know that such and such a supermarket has got a special deal on this, this week? The olive oils are half price”, just little tit bits like that makes you feel part of a community, that you're not in it alone, that there are other people trying to do the same.* Ppt 037, Group 2

*If it became like a team challenge and we had a team goal, that might be quite fun as well to try. And then even to have known what some of the other peer groups were doing would have been interesting as well. That could have been quite motivating, to say “well, the other group across town are doing it and they have achieved this or that,” and you'd have been like “oh, okay … ” So it could have been maybe a wee bit more competitive, and fun.* Ppt 008, Group 3

*Oh, they loved the tasting. The tasting was really good, they did really enjoy that.* PS2, Group 1

*I remember doing the quiz, the fibre quiz, the higher or lower one, and people did really enjoy that and I think people really learnt from that. So, I think having those activities in were quite good and maybe even having more of those activities would have been quite good.* PS2, Group 1

#### Intervention resources

Comments on the intervention resources were somewhat mixed. The majority of participants were positive about the recipe books, with many reporting that they continued to refer to them after the intervention end. The recipe books were considered to be useful for ideas or inspiration, and participants appreciated that the recipes were simple, seasonal, could be prepared quickly, required few ingredients, and showed the nutrient profile of each meal. The recipes were considered to aid the incorporation of some food items specific to the MD, e.g. legumes and seeds, and to be suitable for a range of occasions.

*Especially if people are coming I would take it out and have a go and say look … I would serve it up and wait to see what they said, and say “that's recommended on the Mediterranean diet” and they would all be quite amused about it, thinking this isn't the kind of stuff you eat on a diet. So yes, I do use it*. 018, Group 1

*The recipes and that, we tried a load of them and I enjoyed those too. They helped too. It was just different things, different changes, especially with beans, we wouldn't have had a lot of those and now we're on to bean stews.* 059, Group 3

Not all participants used the recipe books, and improvements were also suggested, to add appeal, variety and take more account of different personal circumstances:

*I tried some of the recipes; they were nice. The biggest problem that I found with them was living on my own, most of them were family orientated; you would make a meal sufficient for four people.* Ppt 037, Group 2

The seasonal shopping lists were not often used. Those that did use the shopping lists reported that they found them useful for ideas and inspiration, or that they found them more useful at the beginning of the intervention to build a stock of cooking ingredients.

*I now have a great stock of spices and things like that, which is great, and I'm really quite proud of them now, and people open the cupboards and go “oh look!” And it's great but that's taken time to build that up.* Ppt 008, Group 3

The feedback regarding the personal planners was varied. Participants mentioned value to the planners for keeping track of goals, returning to goals following failures and for keeping notes, but use of the planners did reduce over the intervention period. Participants reported that they found goal setting to be more useful at the beginning of the intervention as they adopted the MD, than while they were maintaining the diet, but some participants also reported that reduced planner use over time reflected a loss of interest and engagement with the intervention overall.

*Goal setting helped, I find it beneficial to write it down, have it in black and white and try and work to that, because otherwise it's airy fairy, and because in your diet plan you made the goal setting specific, not just an idea that had 10 strands coming off it, it had to be specific, it had to be achievable and something that if I didn't achieve it I could go back and do it again. So that was really beneficial.* Ppt 018, Group 1

*I was lucky, the goals that I set, I very quickly achieved … So it became hard, because you were asked to pick three goals each time, and that was actually a lot, every time then to come up with three new goals. So I would say, probably after seven weeks, I stopped using the goal thing because it just wasn't really … As I say, you were trying to make up things to put it down to say ‘I drink more water, I'll do that.* Ppt 008, Group 3

Negative comments typically referred to either focusing on food or the practice of writing goals in a book in preference to using other media, e.g. a mobile phone app or a post-it note that could be stuck in a prominent position:

*I think over the years I used to write down, if I went on a diet or whatever I wrote down what food etc., but it tended to make me think more of food when I wrote it down, and instead of it making you take less you tended to … it was a focus all the time with regards food. So I suppose I have an aversion to writing that down because of that.* Ppt 021, Group 1

Peer supporters also mentioned challenges to encouraging use of the personal planners:

*I found that was a challenging area, yeah, around the personal planners. The first time, because I suppose it was the first time we'd done it, they were happy enough to set the goals and did set two or three goals each, but when it came to reviewing it and how did it go and do you think that one's routine now, do you want to move on and add something else, that was more challenging. Yeah, it became more challenging over time to continue to use and to make the planners really live the way that you would have wanted them to, and it was something that we would have found when we were asking people to take out the planners, that they weren't really keen. They might have come out of the bag but they, kind of, went under one leg or something.* PS2, Group 4

#### Intervention progression

Difficulties arose as the intervention progressed, as meetings were spaced further apart, or group attendance decreased.

*In the first two months the meetings were every two weeks, I think it may have started to go out to every four weeks towards the end of that two months, and that is why I think it worked early on, because the meetings were regular. Once the meetings started going out to every six weeks, every eight weeks then they just weren't of any benefit, particularly as the group size got smaller and smaller.* Ppt 037, Group 2

*In general, the longer the spell there is, people kind of drift a wee bit and forget, and then if you miss the two monthly meetings then it's four months, I just think in terms of classes and things … you know what happens, if you miss four months then you maybe don't go back.* Ppt 038, Group 4

Smaller meetings were reported as more repetitive and less beneficial, because there was less sharing of information, and some participants found the reduction in numbers demotivating, although one participant discussed a benefit as he/she felt that he/she was doing well by still attending.

*As the numbers started to fall off that probably then became a bit of a de-motivator.* Ppt 008, Group 3

*Some nights you went and maybe there was only one or two, and as I say, there wasn't the same swapping notes and finding out how the people were doing.* Ppt 059, Group 3

Peer supporters also recognised challenges as meetings became less frequent, attendance dropped and meetings became smaller:

*We would have always said that it was working best for us when it was every two weeks, so when it was really regular, and then when we went to monthly, I would say it dropped a wee bit at that point, and then when it went to two monthly, funny, I would have said it dropped again. So I don't know if there's something about the regularity. If people, when they're in a routine, they get something out of it.* PS2, Group 4

*I think probably because the group was so small, as the time went on it was harder to kind of come up with the ideas and to fill the two hours without going over and over everything, and that was probably the most challenging thing.* PS1, Group 2

#### Intervention session attendance

Reasons for missing meetings focused predominantly on personal factors, such as visiting family, looking after family members, having a busy lifestyle, work commitments, holidays or personal illness:

*I've an awful lot of other things on and trying to make it every Tuesday night for a period of so long, I found it difficult sometimes to physically be there, even though I wanted to be there.* Ppt 005, Group 1

Intervention factors were also reported to impact attendance. Participants mentioned not receiving reminders about group meetings, receiving reminders at too short notice to enable attendance and being unable to attend meetings due to a lack of public transport:

*You might have got a text maybe a day or two before you were due to meet again and if you hadn't remembered, it wasn't long enough notice to remind you “oh crikey”, that's tomorrow evening.* Ppt 008, Group 3

Inter-personal issues were also reported to affect attendance, including disappointment with or discomfort around other group members:

*I started off going to the meetings and was quite enthusiastic, because we were all supposed to swap ideas, and I baked a rye bread and brought it into the group, but during the time that I did go, no one else seemed to bring anything, and I kind of felt a little let down at that. Then another girl joined, who lived quite close to me, so we had agreed to go out walking and that we would share the travelling to the meetings, where in fact that didn't happen either. So I felt a bit let down with that as well. I just thought I'll do my own thing, and I kept to the diet and I did do some of the recipes that were in the books, but I stopped going to the group meetings.* Ppt 033, Group 3

Lack of achievement or of perceived intervention success were also offered as reasons for failing to attend by some:

*The group dwindled away, I don't know the reasons for it but I think maybe with the thing “Mediterranean diet” maybe a lot of people thought they were going to be losing a lot of weight rather than maybe change their eating habits, and I don't know whether that might have been part of the reason or something.* Ppt 059, Group 3

#### Final evaluation

Eighteen participants completed the final evaluation questionnaire. These participants agreed that the written literature (mean (sd) out of 4 = 3⋅4 (0⋅6)), planners (mean = 2⋅7 (0⋅9)) and educational topics in the group sessions (mean = 3⋅1 (0⋅8)) were useful; as were options to contact the peer supporters between sessions (mean = 2⋅5 (0⋅7) and to have body weight and blood pressure measurements taken (mean = 2⋅9 (0⋅7)). The level of advice and support provided by the peer supporters was appropriate (mean = 3⋅2 (1⋅1)), and motivating (mean = 2⋅8 (0⋅8) and group locations were considered suitable (mean = 2⋅8 (1⋅2)).

Positive comments also suggested participants had enjoyed the whole intervention and would continue with the MD. Participants also reported sharing their new knowledge among friends and family, providing others with the TEAM-MED resources, and suggested that their participation in the TEAM-MED study had encouraged dietary change in their peers:

*I just think my lifestyle has changed so much now that I don't think that I could go back to that. I don't even remember what it was, because the routine that I have now is so embedded in now that I don't ever see that happening.* Ppt 008, Group 3

*I found people were very interested and very supportive and I would print off copies of the initial book you provided us with and give it to certain people and hope they would use it.* Ppt 018, Group 1

*I have definitely shared a lot of the Mediterranean diet with friends … So I think it's been useful for my family and friends.* Ppt 041, Group 2

However, participants also recognised the importance of personal preferences, the importance of the peer supporter and the necessary synergy between peer supporters and group members:

*I'm not a great group person anyway, not unless it really, really grabs my interest and other people have the same interest. … I, unfortunately, got put in the wrong group for me. I mean, I think, from what I gather, all the other people enjoyed it.* Ppt 047, Group 3

*I would have liked to have been in the dietician led group, …*  Ppt 033, Group 3

*The peer support group could have been co-ordinated better, the facilitator did not take a strong enough lead, initially there was poor communication between the facilitator IE arranging and confirming the times and location of the group. In the later stages when the group was taken over by [name] it became better - more motivated person, by that time unfortunately a lot of people from the group had fell away.* Ppt 008, Group 3

Other suggestions for improvements to the intervention included ensuring continuity in group peer supporters, continuity in group meeting times and locations, improved communication and updates to participants on meetings that they missed.

*Peer group would benefit from continuity in group leader. Also communication could be improved. I was not informed of some of the group meetings. Some I could not have attended because of work commitments. An email explaining what happened in the meetings that I couldn't attend would have kept me up to date.* Ppt 060, Group 3

*Changes to meeting time and place was inconvenient.* Ppt 033, Group 3

### Data collection processes

Comments specifically on the data collection aspects of the pilot trial suggested that too many questionnaires were included as part of the study, and that these were requested too often:

*Not so many questionnaires at one time.* Ppt 065, Group 4

*Far too many questionnaires.* Ppt 047, Group 3

### Reasons for withdrawal

Retention of participants in the peer support intervention was 81%, 70% and 59%, at 3, 6 and 12 months, respectively, showing a gradual decline in participation. At 12 months, one person (out of 6) had withdrawn from Group 1, two people (out of 4) from Group 2, none (out of 6) from Group 3 and seven people (out of 10) from Group 4. Four participants who withdrew took part in follow-up interviews. These participants suggested that reasons for withdrawal largely centred around personal circumstances, such as practical concerns, family commitments, health concerns and time pressures.

## Discussion

This process evaluation was conducted as part of a pilot trial to investigate the effects of a group-based peer support dietary change intervention for encouraging adoption and maintenance of the Mediterranean Diet in a Northern Irish population (TEAM-MED), with the aims of understanding the feasibility of implementing the intervention, how the intervention worked in practice and how it could be improved. Feedback on the peer supporter training and support, data on intervention fidelity and acceptability, data on study processes and reasons for withdrawal were considered. Findings are discussed and suggestions for improvements and developments are provided.

Firstly, suitable peer supporters volunteered to take part in the study, were recruited and were trained, such that the required knowledge, skills and confidence to deliver the intervention were considered to be high following training. Intervention sessions were run by peer supporters, and all eleven sessions for each group were run, with the majority of intervention elements undertaken in each session. Observations of some sessions that found that not all aspects of each session were completed as intended may suggest benefit from interim training or additional support for the peer supporters, as requested by some peer supporters explicitly. Some of the comments from the peer supporters also mentioned reduced enthusiasm for the intervention and reduced confidence in delivering the intervention following delays between training and intervention start; a concern that may also be reduced by increased training and support. The majority of comments from the peer supporters, however, were positive, and suggested benefits from the role. Our findings suggest that the strategies to recruit peer supporters and the provision of training were valuable, such that the implementation of a dietary intervention run by peers, as opposed to professionals, is feasible.

Participants also reported positively on many aspects of the intervention including the engaging nature of the group sessions, the resources provided, the value of the peer supporters, and the supportive and encouraging nature of the group. The session content and materials for the intervention were developed specifically for the study, based on theory and following repeated careful discussion with the target population group^(24,37,38)^. The positive feedback on the intervention suggests benefit from this extensive development.

Positive comments were also offered specifically crediting the peer supporters and their efforts. Furthermore, in recognition of the value of the peer supporters, attendance was notably lower in Group 3 where attendance by the peer supporters was also lower, and dropped considerably in Group 4 where the notes from the observed sessions suggested that the peer supporters themselves may have become discouraged. These findings suggest that the peer supporters, their enthusiasm for the intervention and their interactions with each other and with the group may be key to intervention success.

In some of the more objective measures of intervention acceptability (session attendance, participation and group cohesion), notable differences between the groups were also observed. Session attendance varied between groups but tended to fall in all groups across the 12-month intervention. Attendee participation also varied across the groups and with time, but was lowest in Group 4 where peer supporter support was noted to be least well provided. Group cohesion was good in all groups at months 6/7, but remained high only in Group 1. Withdrawal rates also varied across the groups, ranging from 70% in Group 4, to none in Group 3. Suggested explanations for variations in attendance and participation rates included characteristics of the groups (including the peer supporters) and aspects of the intervention which largely focused on meeting (in)frequency and practical or organisational concerns. No one characteristic of a more cohesive or engaged group could be identified from our small sample of four groups, e.g. based on demographic characteristics; more cohesive and engaged groups instead seemed dominated by similarity between members and between members and the peer supporters. This similarity may be difficult to achieve by chance, but may be enhanced by recruiting group members based on relevant characteristics, such as household size, family situation or lifestyle. Meeting frequency and organisational issues are easily addressed during intervention planning.

The descriptive comments also suggest an interaction between attendance and engagement within the sessions. Fewer benefits were reported from smaller groups and from activities that became repetitive without input from different group members. Group cohesion will also have been affected by session attendance, and bi-directional interactions between attendance, engagement and cohesion are reported in other studies using peer support interventions^(35,36,43)^. If session attendance was largely a result of meeting frequency and practical and organisational concerns, clear organisation of the intervention can be suggested as a key component to a successful intervention. These learnings have also been reported elsewhere^(30,34–36,43–45)^.

The interactive effects of the participants however, and the deteriorations in attendance and engagement may also suggest that the ‘group’ aspect to the intervention may have been lacking to some degree. Many of the positive comments referred to ‘sharing’, ‘tasting food together’ and ‘swapping ideas’, and there were positive reports of some social activities outside of the group sessions. The peer supporters also recognised their own increased difficulties with small groups, negative impacts on their enthusiasm as a result of low attendance and the negative impacts of a lack of social activities within the sessions. The impacts of both positive and negative social support at the personal level are often recognised within dietary interventions^(24–27)^, but comments related to sharing and swapping demonstrate an additional value to the peer support group, and a different type of ‘communal support’ that may be gained based on shared experiences, vicarious learning, commonality and a shared identity, with resultant increases in self-efficacy, confidence and coping^(31–37,45,46)^. This communal support is also notably different from that that may be gained from health professionals, where the provision of accurate, credible information and good role modelling is anticipated^(20,45,46)^. Other studies also recognise the differing and additive support for behaviour change that can be gained from different sources, including lay workers, retired/voluntary health professionals and workplace line managers^(31,34,43,45,46)^. Activities to provide this communal group support, beneficial to both participants and peer supporters, can be suggested to include common goals, collaborative goals, team-building activities and social occasions. Common and collaborative goals are known to enhance commitment to the group and can encourage task progress^(47,48)^; an example here could be a MD score *for the group* to which all members contribute. Activities to discuss strengths and weaknesses of individual group members and to assign clear roles and responsibilities within the group can also encourage commitment to the group and group cohesion^(46–48)^. Based on common goals, commitment and group cohesion, the establishment of a group identity can be facilitative^(49)^. In the intervention scenario, open and supportive discussions of successes and fears, e.g. to add fish to the diet, will allow the provision of novel solutions, build confidence and encourage adherence. In terms of roles and responsibilities, group members could be asked to commit to sending reminders for the next session, providing a dish for everyone for the next session, gaining new knowledge and imparting that to others in the next session. Accountability is thought to be an important aspect of group tasks and their successful attainment^(37,45,46,48)^. Open communication and trust in all group members are also key^(47–49)^. The ongoing development of new peer supporters could be a valuable role for the group, ensuring continuity for the intervention and for dietary maintenance and improved health. Social activities outside of the intervention, such as tea breaks, additional meals or physical activity sessions will also encourage personal relationships and support. The importance of personal connections within peer support groups has previously been emphasised^(35,37,43,46)^. In this respect, use of existing groups^(35,37)^ or the set-up of groups based on relevant baseline characteristics, such as family situation and cooking responsibilities^(46)^, may again be beneficial.

Negative perceptions of the intervention focused largely on specific intervention components, and tended to suggest a role for personal preferences, or were based on personal circumstances, such as work commitments or changes in health. The intervention development work involved consultation with the target intervention group to ensure that preferences were considered, e.g. recommendation for two peer supporters per group, one with dietary experience, one with experience of facilitating groups^(37)^, but a majority decision will potentially never suit everyone, or may seem wise in principle, but in practice, may simply not work. One preference of interest was for a non-group-based intervention. Randomisation of participants is an important part of many intervention tests, but perceptions of choice, free will and autonomy are known predictors of health behaviours^(34,50–52)^, and in a realistic situation, a non-group-based version of the intervention may be more acceptable to some participants. Other researchers also report ‘the futility of a one-size-fits-all approach’ to dietary change^(20, p. 1369)^ and suggest the need for alternative interventions or alternative means of intervention delivery for some participants or at different stages of behaviour change^(20,36,37,43,46)^. Solutions may include the offer of a variety of interventions or intervention components from which individuals can choose. Such an approach may be facilitated through the use of digital technology. Digital interventions can be developed such that personalised interventions can be delivered based on client needs^(53)^, and digital interventions that also include online community groups and fora may provide added social support for those who desire this, without requiring engagement from those who do not. Suggestions for improvements to our intervention included the use of online support groups. Personal preferences, however, will still need to be considered, as will issues in terms of access.

Other interesting negative comments from participants focused on the use of the term ‘diet’ and its negative connotations, the need for recording food consumption, the inclusion of optional body weight measurements and expectations of weight loss that were not met. Similar negative connotations following the use of the term ‘diet’ when referring to MD have also been reported^(26)^, as have expectations of weight loss^(21)^; negative comments that are unfortunate given that weight loss is often a result as opposed to an intended goal of the MD^(20)^. Body measurements were included in the intervention as a behaviour change technique referred to as ‘biofeedback’^(54)^, and despite ideas from participants during intervention development that this would be motivating^(37)^, in hindsight, inclusion of the weight measurements within the group sessions may have provided an unintended focus, and may in fact have been demotivating when little change was seen. It is possible that the benefits of biofeedback may have been better achieved through the use of biochemical markers or blood parameters that more closely reflect the intended outcomes of the MD, e.g. pinprick lipid or cholesterol profiles, but these measurements are more intrusive than taking weight measurements. A focus on MD as a dietary pattern and lifestyle choice, without reference to ‘diet’ furthermore, may be beneficial. Use of an alternative term, such as the Mediterranean Dietary Pattern or a Mediterranean Lifestyle Pattern, and consideration of other aspects of the MD, such as consumption of fruits and vegetables at every meal, eating slowly, eating in social settings and a moderate amount of physical activity may have also removed the focus on ‘diet’^(4,5)^.

Some clear recommendations for developing a peer support intervention to encourage the adoption and maintenance of a Mediterranean Diet can be gained from our findings, as given in Box 1.

Box 1 Recommendations for developing a peer support intervention to encourage the adoption and maintenance of a Mediterranean Diet.

- Development of a comprehensive and well-structured intervention will provide structure to the sessions, while imparting necessary knowledge and skills.
- Thorough initial and continued training of the peer supporters prior and during intervention delivery will ensure initial and continued knowledge, confidence and enthusiasm.
- Clear organisation of the sessions both in terms of logistics and content, and clear commitment from the peer supporters will encourage attendance and engagement from participants.
- Meetings at a frequency of one or more per month may enhance participant attendance, engagement and group cohesion.
- More than one peer supporter per peer support group will ensure continued intervention provision, organisation and commitment, particularly in unforeseen circumstances.
- Activities to enhance support for the ‘group’, e.g. the establishment of common goals, clear roles and responsibilities for each individual, discussion of strengths and weaknesses will enhance group cohesion, attendance and engagement.
- Activities to reinforce additional similarities between group members, e.g. social activities, and/or the set-up of groups based on relevant personal characteristics will also enhance group cohesion.
- Collaboration, knowledge-generation and sharing responsibilities will encourage ongoing and sustainable implementation and success.
- Consideration of individual preferences will enhance uptake and maintenance of the intervention in realistic scenarios.
- Avoiding a focus on ‘diet’ and body weight, and consideration of wider aspects of the MD ‘lifestyle’ may broaden and strengthen the appeal of an MD intervention.

The peer support intervention was originally designed as an intervention that could cost less to run than a dietitian-led intervention, and comparable effects were found in the trial, in many dietary and health outcomes^(40)^. If suggested improvements can further enhance its effectiveness, the possible benefits of the peer support intervention would be increased. Positive impacts for the peer supporters were recognised, and other researchers report benefits from the supporter role^(29,30)^. Some studies however, also suggest detriments^(36,43)^, and adequate training and support for the supporters is recommended^(30)^. A cost-benefit analysis of the peer support intervention for dietary and cardiovascular health, following assessment in a trial of suitable power in comparison with existing offers, would clearly be of value. The peer support intervention has already been developed, thus much of the cost of this intervention has already been covered, although consideration for the training, time and ongoing support for peer supporters will be important^(30,36,43)^.

Added benefit may also be gained by allowing individuals to choose the intervention that they undertake. Some participants enjoyed the group-based nature of the peer support intervention, while others stated a preference for less interaction. While not enabling the use of a randomised controlled trial to test effectiveness, autonomy or an element of choice is an important part of theories of motivation^(51,52)^, and providing individuals with this choice, allowing a match between support sought and support provided, may improve outcomes.

The strengths of this process evaluation lie in the variety of data collected from questionnaires, observations and a considerable number of interviews, from both participants and peer supporters. Many of our findings are also not specific to the Mediterranean Diet aspect of the intervention, could apply to other peer support interventions for dietary change, and have resulted in recommendations that may apply to a range of behaviour change interventions using peer support. The evaluation is limited by our reliance on the data offered from a limited set of volunteers, to researchers with invested interest, and thus some reporting bias can be suggested, although this is unlikely to be unidirectional. The pilot trial from which the data were gained, was limited in sample size and population group. This was intentional as a first step to testing this intervention for health benefits, but other aspects of the intervention may need to be considered, or may be more important, in other population groups.

## Conclusion

In conclusion, this process evaluation demonstrates the feasibility of developing and implementing a peer support intervention for dietary change, that was both positively received and undertaken by members of the target population group. Key components for success were considered to be the intervention and session materials; the peer supporters, including their training; and the supportive nature of the group meetings. Ongoing peer supporter training and support, increased interactive and social activities, including group and collaborative goals, and clear organisation ensuring good attendance may further enhance the successful nature of these types of intervention. Some consideration of personal preferences may also improve outcomes.

## Supplementary material

For supplementary material accompanying this paper visit https://doi.org/10.1017/jns.2023.2. click here to view supplementary material
