# Supplementary material for: A peer support dietary change intervention for encouraging adoption and maintenance of the Mediterranean diet in a non-Mediterranean population (TEAM-MED): lessons learned and suggested improvements
Source: J Nutr Sci. 2023 Jan 30;12:e13. doi: 10.1017/jns.2023.2 (PMC9947623; doi:10.1017/jns.2023.2)
Supplement: Supplementary file 1 [file S2048679023000022sup001.docx]

**SUPPLEMENTARY MATERIALS**

**Section: Methods: Interview Topic Guide**

- What were your main reasons for taking part in the TEAM-MED study?
- What did you enjoy most about the group programme? Why do you think this was the case? And what was least enjoyable or useful?
- How did you find the atmosphere and mix of people and peer supporters in the group?
- In what ways were the group able to support one another to make changes towards a MD?
- What other types of support helped you to make changes to your diet in the past 12 months? (From friends, family, other groups?)
- How useful was the personal planner to set your dietary goals and track your progress? Did you use it much in the past 12 months? If not, why do you think this was the case?
- Overall, what do you think we could or should do to improve the group meetings if they were offered to the public?

**Section: Methods: Session Evaluation Checklist**

Includes 23 elements of each intervention session that were expected from the peer supporters (unshaded), and 11 elements of the sessions which reflect participation from the attendees (shaded). One item (number 24, heavily shaded) referred to the research team and was not considered further.

| Date: _ _/_ _/_ _ _ _ | Researcher: ______________________ |
| --- | --- |
| Group number: _ _ | Session: _ _ |
| Number of Peer Supporters in attendance: _ | Discussion Topic: __________________ |
| Number of members expected: _ _ | Number of members in attendance: _ _ |

If the statement provided is correct, tick in the ‘yes’ column. If the statement is false, tick in the ‘no’ column and provide a **brief explanation** in the comments section. **Shaded comment sections must be completed** to justify the answer of that section.

| Statement | Yes | No | Comments |
| --- | --- | --- | --- |
| Social Support & Engagement | | | |
| 1. Peer supporters welcome group members.▫ |  |  |  |
| 1. Peer supporters initiate discussion about dietary progress. |  |  |  |
| 1. Peer supporters initiate discussion about topic using open ending questions. |  |  |  |
| 1. All group members contribute to discussion. |  |  |  |
| 1. All group members contribute equally to discussion. |  |  |  |
| 1. Peer supporters encourage** group members in their progress. |  |  |  |
| 1. Challenges to changing or maintaining dietary goals are identified. |  |  |  |
| 1. Options to solve problems are discussed. |  |  |  |
| 1. All challenges are resolved with solutions. |  |  |  |
| 1. Group members offer practical help to each other. |  |  |  |
| 1. All group members engage with their personal planners. |  |  |  |
| 1. All group members review their dietary goals. |  |  |  |
| 1. All group members form new dietary goals. |  |  |  |
| 1. All group members are open in discussion of their personal goals. |  |  |  |
| 1. Peer supporters encourage group members to meet between sessions. |  |  |  |
| 1. Peer supporters thank the group and close the meeting. |  |  |  |
| 1. The nature of discussion is generally positive. |  |  |  |
| 1. Discussion is generally focused throughout the meeting. |  |  |  |
| 1. Both peer supporters contribute equally. |  |  |  |
| 1. Active listening skills are demonstrated by all attendees. |  |  |  |
| Format & Environment | | | |
| 1. Attendance register is taken. |  |  |  |
| 1. Peer supporters arrive at least 10 minutes prior to start time. |  |  |  |
| 1. Peer supporters are organised with all necessary resources*. |  |  |  |
| 1. Researcher provides seasonal Mediterranean dish if appropriate. |  |  |  |
| 1. The environment is appropriate to facilitate the group meeting.▪ |  |  |  |
| 1. Previous queries are answered if required. |  |  |  |
| 1. Peer supporters introduce the session topic. |  |  |  |
| 1. All group members have their blood pressure measured. |  |  |  |
| 1. All group members have their weight measured. |  |  |  |
| 1. Health measurements are taken accurately† and recorded. |  |  |  |
| 1. All partaking group members record their own measurements in their personal planners |  |  |  |
| 1. Peer supporters summarize key learning points from the session. |  |  |  |
| 1. Questions for study team are recorded. |  |  |  |
| 1. Peer supporters inform group members of the next group meeting; subject and time. |  |  |  |
| 1. The session concludes on time. |  |  |  |

1. What is your overall impression of group cohesiveness?

| Low |  |  |  |  |  |  |  |  | High |
| --- | --- | --- | --- | --- | --- | --- | --- | --- | --- |
|  |  |  |  |  |  |  |  |  |  |

1. What is your overall impression of the peer supporters attitude and approach to the group?

Additional Comments (record anything brought in by peer supporters or group members, e.g. food or presents, reasons for non-attendance if available, previous between session contact if discussed, any interesting outcomes or difficulties faced by participants)

▫All participants are welcomed by name, briefly asked about their wellbeing and offered refreshments.

*Necessary resources: attendance register, recipe books for season, pens, paper, Mediterranean diet poster, ground rules poster, weighing scales, blood pressure monitor, refreshments and TEAM-MED study health measurement record

**Encouragement: recognition of success, words of congratulations, support for participants experiencing difficulty

▪The environment should be a comfortable temperature, there should be sufficient chairs, chairs should be set out in a circle or semi-circle, there should be sufficient lighting and refreshments should be laid out.

†Blood pressure must be measured as per protocol: ensure group member sits in a quiet room for at least 5 mins, measure brachial blood pressure using an oscillometric technique, measure blood pressure in the right arm, take three consecutive readings over 5 mins, calculate a summary blood pressure from the 2^nd^ and 3^rd^ reading.

**Results: Example Quotes**

**Section: Peer supporter training and Support**

**Training**

*“It was good. The resources that we had were very good, the folder and the props that we had were very good, and the training went through all of that.”* PS3, Group 2

*“It was very a good mixture of theory based with the resources, the paper resources that you had to use with each of the groups. It was all very clearly laid out and there was a good combination of the theory, actually doing the workshops you’d be getting the group to do and then feeding back what you thought of the workshops and the processes. It was very interactive sort of learning through doing and learning with your other peer supporters and getting to know them as well.”* PS1, Group 4

*“I really enjoyed, really did enjoy the two days. I learned an awful lot, probably in quite a short period of time. But, no I really did enjoy that and knew, picking up things that I maybe, wee things that I had thought, maybe wee myths and things that I had thought, so they ironed all that out for me.”* PS2, Group 2

*“I suppose sometimes maybe it was just some of the topics maybe were a bit light in detail and you found you had to do a bit more research yourself and reading beforehand just to make sure that... just go and Google wholegrain or whatever, just to get a bit more information, a bit of depth behind, because, at the end of the day, you have to spend two hours with the group talking about it and you maybe felt that you needed a wee bit more information in the pack or on each topic, it would have been helpful.”* PS1, Group 4

*“There was a big time delay between when we were trained and then we started as a peer supporter, and even just I did have, I think it was an hour, an hour and a half where I met … my other peer supporter. We met for the first time and we went through a few things. I suppose you were very motivated after, I think, it was two days of training as a mentor, and then it was really like disappointing, or frustrating maybe, that the length of time it took from doing the training, to starting with the group, because you were all motivated and going ‘right, yes; I can see what I want to do here and let’s get started’. It’s going ‘all right, hold on, I’ve forgotten everything that I was taught or learnt through the training.’ So there is a nearly, a wee bit of apprehension and nervousness on your path. But once you actually get into the routine of it and the role of it, and actually get your head around, just get back up to speed with the manual, it’s all there and it was very easy to follow. So you kind of just got the confidence back and the motivation back once you started and once you got to know your groups. So it was a wee bit frustrating but once you got started you sort of forgot the big delay, you know.”* PS1, Group 4

*“Once I did start I had to really reread everything, which wasn't a bad thing. But I think I would have liked maybe a quicker start from the initial training”* PS2, Group 2

**Final Evaluation**

*“I suppose that’s what motivates me a lot, to try and help more people be healthier, live healthier lifestyles. So yeah, it was very positive. We met some very interesting people, and I suppose I learnt quite a bit about Mediterranean eating and Mediterranean lifestyle and tried to incorporate a bit of it.”* PS1, Group 4

*“I always like meeting people and getting to know people, and also knowing the content. I enjoyed just getting really in-depth with the knowledge of the Mediterranean diet. So all of those things I’ve been involved in before, in working with groups, and that was what I enjoyed about it, was working with the people and seeing if I could make any changes or improvements and what would be the outcomes.”* PS2, Group 4

*“I think just meeting the group and building up a relationship with them and just seeing how they were progressing and discussing with them their problems as well as their successes and their failures, really. And their honestly, I think, was very... it was lovely to see, yeah.”* PS1, Group 1

*“I suppose seeing them get to know each other as a group and to see how their individual journeys, how they kind of progressed and the changes that they were able to implement. So that was really interesting. And just to see, over the course of the year, how their enthusiasm didn't really wane at all, like they stayed really, really, sort of really keen and really focused, which, it was really nice to see that, and that was the most interesting aspect for me. It was nice just seeing how the group evolved and how they interacted with each other as well and how we - it was very, like it was very relaxed and it was a very supportive group, and it was nice to see that develop.”* PS2, Group 1

*“I think at the start when we had the four members it was seeing the success that they were getting on the programme, and the ideas that they came with, and then the enthusiasm. As the groups got obviously it's obviously got much, much smaller, but it's still nice to have the contact and know that people will go on to carry it on and hopefully the health benefits will be of use to them.”* PS1, Group 2

*“The most enjoyable part was just seeing the enthusiasm of the group. Sometimes only a couple of people turned up, but there was never a negative evening with them, they were always very positive, which was really nice to see.* PS3, Group 3

*“The first meetings, there was just that many questions it was like a firing squad [laughing]. You were trying to manage it, and it was good from that respect. But I remember on two or three of early meetings suggesting right, will we break now for tea and coffee? They were "no, no, just keep going with this"* PS2, Group 4

*“It would have been nice to have a bit of support maybe from another mentor, especially, funny enough, the last session, which is the one I would have thought would be the easiest, but that was the one that you had to recap everything and because I had forgotten my folder, I forgot the questions [laughing] But it would have been nice to have a little bit more support on that.”* PS3, Group 3

**Section: Intervention Acceptability**

**Intervention sessions**

*“It was good just to learn about the difference between wholegrain and all those sort of things, because sometimes you hear all this talk and you feel a bit silly because you don't understand. It wasn't like that; any time that you were in the group you didn't feel as if you were asking a silly question.”* Ppt 012, Group 1

*“If you said ""oh I've tried that,"" or especially if somebody had achieved a goal of, like I say, when he had said he had walked, and we were all ""oh that’s fantastic!"" and ""imagine doing that!"" Or equally too, if somebody had tried something for the first time, even if they didn’t like it, we’d say ""well at least you tried it."" So it would have been very encouraging.”* Ppt 008, Group 3

*“Well, one of the ladies had brought in some particular type of bread one day and said you know, you can get this here and there, and I hadn’t taken a note so I contacted her and she told me again where it was. But not only that, her husband was going up, because it was up in the xxx direction, and she brought me in a loaf the next week.”* Ppt 005, Group 1

*“a few more ice breakers to get people going and a wee bit more engaged, and I'm thinking from memory, because I don’t really know even at the start if it was explained what the purpose of the peer support group was and about what your responsibility is within that group.”* Ppt 008, Group 3

*“it could either be an ice breaker or something...an activity is booked maybe three times during the year or something like that, that would help people socialise more, because it was a very ...it wasn't a social activity.”* Ppt 033, Group 3

*“I think, for the type of group that it was, the interest was a personal interest for each individual. So although it was people following a Mediterranean diet, I think the cohesion wasn't a group. Each person had their own individual interest at heart, rather than with other people.”* Ppt 033, Group 3.

*“Putting the responsibility back to the people in the group to bring something forward. So even a ‘show and tell’ thing of making people say "look, it’s your turn next time to bring something”. So the onus is on them, because it is peer support so we’re all supposed to take responsibility.”* Ppt 008, Group 3.

*“Signing up to it and saying 'I really want you to make the commitment here. It's all right for you, you might be getting on well with it, but there could be somebody else here who's really struggling and maybe a wee bit of motivation and support.”* Ppt 008, Group 3

“*If it became like a team challenge and we had a team goal, that might be quite fun as well to try. And then even to have known what some of the other peer groups were doing would have been interesting as well. That could have been quite motivating, to say ""well, the other group across town are doing it and they have achieved this or that,"" and you'd have been like ""oh, okay..."" So it could have been maybe a wee bit more competitive, and fun.”* Ppt 008, Group 3

*“WhatsApp would be good because it's free, and you could take photographs of what you're eating, put it around your group and say ""look at me tonight"". I think that would be quite good and it would be good fun and sort of an element of yes I'm actually doing this, I'm not just talking about it, I'm actually eating this tonight."" ""Look at me; I'm eating fish!"" That sort of thing would be good. I think I would have enjoyed that.”* Ppt 041, Group 2

*“a Facebook page or something like that that's relatively quick and easy to set up, that again your sharing tips, hints, successes, ""did you know that such and such a supermarket has got a special deal on this, this week? The olive oils are half price"", just little tit bits like that makes you feel part of a community, that you're not in it alone, that there are other people trying to do the same.”* Ppt 037, Group 2

*“They responded most positively to the resources that they were given, the materials that they were given. For example, the recipe books, the shopping lists, the information sheets on the different topics that were covered, and again, we responded with the answers to their questions from the previous week. The other, I suppose, very social thing that does pull people together is when they’re tasting food together and getting people to sample the small packs of nuts we took in.”* PS1, Group 4

*“Oh, they loved the tasting. The tasting was really good, they did really enjoy that.”* PS2, Group 1

*“I remember doing the quiz, the fibre quiz, the higher or lower one, and people did really enjoy that and I think people really learnt from that. So, I think having those activities in were quite good and maybe even having more of those activities would have been quite good.”* PS2, Group 1

**Intervention Resources**

*“Goal setting helped, I find it beneficial to write it down, have it in black and white and try and work to that, because otherwise it's airy fairy, and because in your diet plan you made the goal setting specific, not just an idea that had 10 strands coming off it, it had to be specific, it had to be achievable and something that if I didn't achieve it I could go back and do it again. So that was really beneficial.”* Ppt 018, Group 1

*“It was very good, yes, because I was able to look over the previous weeks and days and that. Certain events occurred where you slipped back because of having to attend functions where there was big meals late at night where there was no choice of menu. But all in all, you tried to adapt the next few days after any of the fall backs.”* Ppt 003, Group 3

*“I was lucky, the goals that I set, I very quickly achieved... So it became hard, because you were asked to pick three goals each time, and that was actually a lot, every time then to come up with three new goals. So I would say, probably after seven weeks, I stopped using the goal thing because it just wasn’t really... As I say, you were trying to make up things to put it down to say 'I drink more water, I'll do that.”* Ppt 008, Group 3

*“So maybe even to work it back the other way that, rather than set the goal, is there some sort of system that, without making it too childish, but ""there’s a star this week because you've managed to continue to do that."" So it's more like ""I’ve maintained it."* Ppt 008, Group 3

*“I wasn't going to the meetings so therefore I wasn't writing it up. Once you miss a month, by the next month you don't bother, and then by the next you don't bother at all. Just lack of organisation and a bit of laziness.”* Ppt 038, Group 4

*“although you were encouraged at the end of each one to write them out, sometimes we ran out of time and maybe only got to two people in the group and we wrapped it up and off we went. I did find it a bit laborious and probably whenever I went for one of my study groups at the Royal, or one of my analysed study/appointment days, I don't even think we were doing it particularly well, because we didn't have enough goals and we didn't maybe have it detailed enough as to what we should be.”* Ppt 041, Group 2

*“when you wrote them down they became very dead and I think if we could have found a way, and I don’t even know if writing them in the way that we wrote them in the book, even if it had been something that you could have then stuck up on your fridge.”* Ppt 008, Group 3

**Intervention progression**

*“In the first two months the meetings were every two weeks, I think it may have started to go out to every four weeks towards the end of that two months, and that is why I think it worked early on, because the meetings were regular. Once the meetings started going out to every six weeks, every eight weeks then they just weren't of any benefit, particularly as the group size got smaller and smaller. It just so happened that that also coincided with me not being able to attend as well. It snowballed, as it were, that the reasons for not following it through just got a bit bigger each time.”* Ppt 037, Group 2

*“I think it would have benefited me more had it been every month rather than it going on to a two monthly.”* Ppt 038, Group 4

*“It was when we didn't meet for a month or six weeks, I found then that it was hard to get the momentum to go back again.”* Ppt 041, Group 2

*“I think once a month. If you could stick to the first Monday in the month, or the second, or the first Tuesday in the month that would be good, then you knew exactly where you were.”* Ppt 041, Group 2

*“Maybe start off at the two weeks and then opened it up to a month. Not go any further than that, keep it monthly.”* Ppt 052, Group 3

“*It was inconsistent and I... well, I just got lost with it. I did fall away and I'd lost interest in going to the meetings. I didn't lose interest with the diet.”* Ppt 033, Group 3

*“In general, the longer the spell there is, people kind of drift a wee bit and forget, and then if you miss the two monthly meetings then it's four months, I just think in terms of classes and things...you know what happens, if you miss four months then you maybe don't go back.”* Ppt 038 Group 4

*“As the numbers started to fall off that probably then became a bit of a de-motivator.”* Ppt 008, Group 3

*“Some nights you went and maybe there was only one or two, and as I say, there wasn’t the same swapping notes and finding out how the people were doing.”* Ppt 059, Group 3

*“You did have that where you were thinking 'well, people aren’t sticking at it but at least I am.' Do you know what I mean?”* Ppt 052, Group 3

*“It became, "well I've got to go out on a Tuesday night for a couple of hours to go over the same things with the same two people," who are not really members of the group, they're the facilitators, so you are not getting any further feedback from people who are trying to do the same as you.*” Ppt 037, Group 2

*“A lot of them seemed to drop off in between months and if you missed a month or a session, it might be four months before you had another session, which seems a very long time.”* PS3, Group 2

*“We would have always said that it was working best for us when it was every two weeks, so when it was really regular, and then when we went to monthly, I would say it dropped a wee bit at that point, and then when it went to two monthly, funny, I would have said it dropped again. So I don’t know if there’s something about the regularity. If people, when they’re in a routine, they get something out of it. Maybe that was something to do with it, the timing, just the drop down to the four weeks, and we also had a change of venue.”* PS2, Group 4

*“At the beginning, a meeting every couple of weeks, that was good from the point of view kind of getting to know the participants well. Then as it went on and it was more spread out, especially when it was six weeks to two months, in a way, I think you lost that continuity, to some extent, and it might have been better maybe, on a more regular basis, maybe once a month over the whole period of time.”* PS1, Group 1

*“There was contact just to remind people of the sessions and ask them if they’d any questions. “Just like ""we’re meeting; we’ll see you there."" Mostly people got back to us at the start and then it sort of, you would have sent your text and then it was like ""I’m not getting anybody so I’ll phone people,"" and you weren’t getting really any response over the phone. And if some people couldn’t attend, if people couldn’t attend, when they did let us know, there was specific people that would say ""I’m sorry, I have to work,"" or I have this on or I’ve that on, and if it was a week where we were giving out the recipe books, instead of the person missing out on the recipe book, we would have posted it out to them, which we were happy to do but then we sort of thought, after a while, 'aren’t we just making it easy for them not to attend the sessions, but they still get the materials?' So, I don’t know.”* PS1, Group 4

*“I think probably because the group was so small, as the time went on it was harder to kind of come up with the ideas and to fill the two hours without going over and over everything, and that was probably the most challenging thing.”* PS1, Group 2

*“We were really, at that stage then we were just repeating, you know, and saying the same things that we'd said, and because the group was so small they knew quite a wee bit as well, you know, so they had sort of grasped it really quickly and were getting on with it really well.”* PS2, Group 2

**Final Evaluation**

*“You say to people I’m doing that for a year now, and they say ""a year’s a long time."" But it flew in and it has changed my whole mindset regarding food.”* Ppt 059, Group 3

*“I just think my lifestyle has changed so much now that I don’t think that I could go back to that. I don’t even remember what it was, because the routine that I have now is so embedded in now that I don’t ever see that happening.”* Ppt 008, Group 3

*“I think it's a matter of trying to maintain what I'm doing. As I say, it's not 100% but it's a big change from the way I used to eat.”* Ppt 021, Group 1

“*People went out of their way to help and, as I say, when I was out with the walking groups my friends used to say ""oh he’s on the Mediterranean diet,"" and then people used to say ""what does that entail?"" started to ask me. So there was a lot of questions and all about it and, as I say, I enjoyed telling people.”* Ppt 059, Group 3

*“I found people were very interested and very supportive and I would print off copies of the initial book you provided us with and give it to certain people and hope they would use it.”* Ppt 018, Group 1

*“I have definitely shared a lot of the Mediterranean diet with friends… So I think it's been useful for my family and friends.”* Ppt 041, Group 2

*“ [I] would have liked to have been in the dietician led group”* Ppt 033, Group 3

*“I’m not a great group person anyway, not unless it really, really grabs my interest and other people have the same interest. … I, unfortunately, got put in the wrong group for me. I mean, I think, from what I gather, all the other people enjoyed it.”* Ppt 047, Group 3

*“Peer group would benefit from continuity in group leader.”* Ppt 060, Group 3

*“The peer support group could have been co-ordinated better, the facilitator did not take a strong enough lead, initially there was poor communication between the facilitator, arranging and confirming the times and location of the group. In the later stages when the group was taken over by xxx it became better - more motivated person, by that time unfortunately a lot of people from the group had fell away.”* Ppt 008, group 3
